# Extended Duration of Anti-HEV IgM Seropositivity in Asymptomatic Blood Donors: Implications for Transfusion Safety

**DOI:** 10.3390/v18010088

**Published:** 2026-01-08

**Authors:** Jan Kempski, Maria Mader, Samuel Huber, Sven Peine, Jens Hiller, Julian Schulze zur Wiesch, Sven Pischke

**Affiliations:** 1 Department of Medicine, University Medical Center Hamburg-Eppendorf, 20246 Hamburg, Germany; 2 Mildred Scheel Cancer Career Centre HaTrics, University Medical Center Hamburg-Eppendorf, 20246 Hamburg, Germany; 3 Institute of Transfusion Medicine, University Medical Center Hamburg-Eppendorf, 20246 Hamburg, Germany; 4 Deutsches Zentrum fur Infektionsforschung eV Standort Hamburg-Lubeck-Borstel-Riems, 20246 Hamburg, Germany

**Keywords:** Hepatitis E Virus, epidemiology, serology, blood transfusions, HEV transmission

## Abstract

Infection with Hepatitis E Virus (HEV) is often asymptomatic but can also lead to chronic infection in immunosuppressed individuals. Although fecal–oral transmission of HEV is well established, transmission by blood transfusion has also been reported. Here, we studied HEV seroprevalence in a cohort of 1000 blood donors (50% male, age 18–73 years, mean 35 years) at the University Medical Center Hamburg–Eppendorf in Germany. We found a seroprevalence of anti-HEV IgG of 16.6%. Interestingly, 1.3% of the blood donors had positive IgM serology despite testing negative for HEV by polymerase chain reaction (PCR). Analysis of preceding and follow-up samples showed persistence of IgM antibodies for up to seven months in asymptomatic individuals. In eight individuals, anti-HEV IgM positivity persisted for 0 to 7 months (median 2 months), as confirmed by testing stored samples. This study demonstrates that anti-HEV IgM positivity can persist for more than six months in individuals who had neither clinically overt hepatitis E nor a viremia duration that would allow PCR positivity to be detected.

## 1. Introduction

Hepatitis E Virus (HEV) infection is one of the most frequent causes of viral hepatitis worldwide. It has been estimated that around 20 million people are infected each year [1]. In industrialized nations, most autochthonous HEV genotype 3 infections remain asymptomatic and resolve with spontaneous clearance of the pathogen [2]. However, HEV infection can also result in chronic disease in immunocompromised individuals [2]. HEV genotype 3 infections in the Western World are mainly transmitted zoonotically via the consumption of raw or undercooked pork [2]. However, infections by blood transfusions from viremic individuals have also been reported [3,4]. In Germany, approximately 1/800 blood donors test positive for HEV viremia by PCR [4]. Previous studies have identified that screening for infectious blood donors should be performed by PCR, since both IgM and IgG antibodies are inadequate to separate HEV viremic from non-viremic donors [5]. In addition to the rate of positivity for HEV testing by PCR, which indicates ongoing HEV infection, and the rate of anti-HEV IgG positivity, which demonstrates previous HEV exposure, little is known about the value and the duration of persistence of anti-HEV IgM positivity in IgM testing in asymptomatic persons with HEV in an HEV genotype 3 endemic region. Mansuy et al. reported, in a nationwide French study of more than 10,000 blood donors, an anti-HEV IgG seroprevalence of 22% and an anti-HEV IgM seroprevalence of 1% [6]. In contrast with these data from France, only 5% of 1559 Scottish blood donors tested anti-HEV IgG-positive, and none tested IgM-positive [7]. In a small Italian cohort of 170 blood donors, approximately 10% tested positive for anti-HEV IgG and approximately 2% for anti-HEV IgM [8]. In line with this observation, approximately 2% of blood donors from Switzerland tested positive for IgM [9].

To investigate a larger dataset, a meta-analysis has been performed. The dataset of this meta-analysis contains 206 datasets with 225,328 blood donors. A European anti-HEV IgG seroprevalence rate of approximately 19% for the anti-HEV IgG Wantai test has been calculated [10].

To complete the epidemiological data on anti-HEV IgM seroprevalence among blood donors in Germany, the present study investigated the frequency of anti-HEV IgG and IgM positivity and the duration of IgM positivity in blood donors at the University Medical Center Hamburg–Eppendorf, the largest university hospital in Northern Germany.

## 2. Materials and Methods

Anonymized samples from 1000 unselected blood donors who tested negative for HEV as part of the mandatory PCR test in Germany were tested for anti-HEV IgG and IgM (Wantai test, Beijing, China). These consecutive blood donors from our hospital were recruited between 16 March and 26 March 2020. In Germany, all blood products have been legally tested for HEV by PCR since January 2020. It has already been described that, in Hamburg, Germany, around 1/800 blood donors tests PCR positive, using this technique [4]. Testing was performed in pools of 24 samples using the Roche Cobas, according to the manufacturer’s instructions (Roche Mannheim, Mannheim, Germany). The PCR has an LOD (Lower-Limit-of-Detection) of 432 IU/mL (pool of 24 blood donors, 10 IU/mL for single sample testing). Prior to donation, all blood donors were screened for symptoms of illness, and only individuals who were asymptomatic and reported feeling well were eligible to donate. Therefore, the cohort comprised asymptomatic blood donors. A formal vote by an ethics committee for anonymized testing of residual material was not required under German regulations (Hamburgisches Krankenhausgesetz (17 April 1991, HmbKHG) §12).

## 3. Results

### 3.1. Seroprevalence of HEV IgG in a Blood Donor Cohort

The cohort consisted of 1000 blood donors, 503 (50%) of whom were male. Ages ranged from 18 to 73 years, with a mean of 35 years (standard deviation 14 years). ALT ranged from 9 to 245 U/mL, with a mean of 25 U/mL (standard deviation 25 U/mL).

**Figure 1:**
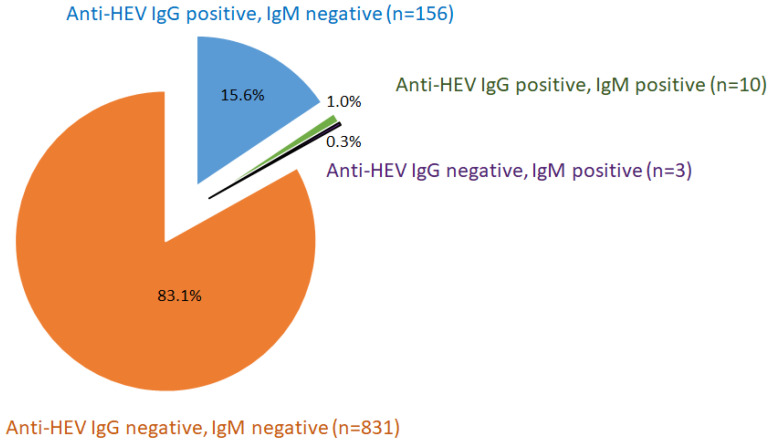
Distribution of anti-HEV IgG- and IgM-positive and -negative subgroups of asymptomatic blood donors.

The seroprevalence of anti-HEV IgG antibodies was 16.6% of the studied population (166/1000) (Figure 1). Unsurprisingly, seroprevalence increased with age: 13.7% in people under 35 years; 32.1% in people over 55 . There was no statistically significant difference between males and females, although the male gender was by trend associated with a higher seroprevalence (Chi-square test 18.7% vs. 14.2%, *p* = 0.09).

### 3.2. Persistence of Anti-HEV IgM in Previously Asymptomatic Blood Donors with HEV

A rate of 1.3% of blood donors tested positive for anti-HEV IgM antibodies (13/999; one out of 1000 missing) (Figure 1). In the group of IgM-positive individuals, eight were male (62%). Ages ranged from 20 to 70 years, with a mean of 37 years (standard deviation 18 years). ALT ranged from 12 to 30 U/mL, with a mean of 20 U/mL (standard deviation 6 U/mL). Neither ALT, age, nor sex differed significantly between IgM-positive and -negative individuals (Mann–Whitney and Chi-square tests).

Since many people are regular blood donors, we were able to test stored serum samples from the preceding and succeeding donations for anti-HEV IgM. Indeed, follow-up serological analysis was possible in 9 of the 13 IgM-positive individuals (with no follow-up donation in the remaining 4). Furthermore, HEV serology was also analyzed in the preceding blood donation in 7 of those 13 individuals (Table 1). Overall, our findings indicate that at least three people remained IgM-positive for three consecutive blood donations (Table 1) over a period of 4–7 months. It is worth noting that Person 7 and Person 9 (Table 1) were IgM+ only at the index blood donation and were both IgM-negative or borderline and IgG-negative in both the preceding and follow-up blood donations. Except for those two people with questionable HEV IgM positivity at the index blood donation, we did not find any person in whom we could observe both the appearance and loss of IgM antibodies. Thus, the persistence of IgM antibodies over even longer time frames (above 4–7 months) remains possible.

### 3.3. Estimation of Anti-HEV IgG and IgM Titers Basing on Anti-HEV OD Values

Because no approved anti-HEV test exists that accurately measures anti-HEV IgG and IgM titers, we collected ELISA OD values as a correlate of the titer. In the total cohort, IgG OD values ranged from −0.121 to 15.568, with a mean of 1.389 (standard deviation 3.513). IgM values ranged from −0.164 to 4.994, with a mean of 0.051 (standard deviation 0.262). In the subgroup of IgM-positive individuals (*n* = 13), anti-HEV IgG OD values ranged from −0.068 to 15.016, with a mean of 10.087 (standard deviation 5.966), and anti-HEV IgM values ranged from 1.046 to 4.994, with a mean of 1.862 (standard deviation 1.127). Negative OD values occur when patient sera have lower OD values than the negative control. This is due to calibration.

There was no significant correlation between anti-HEV IgM OD values and age, but anti-HEV IgG OD values and age were significantly correlated (R = 0.095, *p* = 0.003). Furthermore, anti-HEV IgG and IgM OD values showed a relevant correlation (R = 0.370, *p* < 0.001).

**Figure 2:**
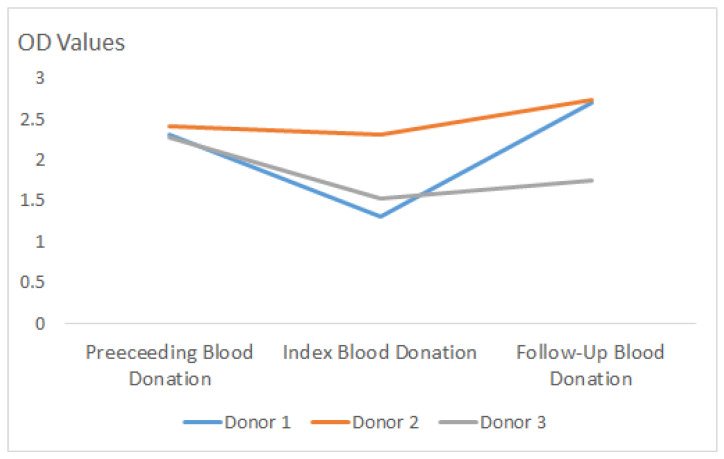
Course of IgM OD values of 3 repeatedly anti-HEV IgM-positive individuals.

Patients 3, 5, and 6 repeatedly tested anti-HEV IgM-positive for seven, four, and seven months after initial positivity. An analysis of their course of OD values did not show a sustained decrease in anti-HEV IgM titers (Figure 2).

### 3.4. Short Duration of HEV Viremia

We previously reported the clearance of HEV-RNA with a median time of 57 days in asymptomatic blood donors with HEV [4]. In line with that report, we identified one person (Person 4) who was IgM- and IgG-positive at the index blood donation and the follow-up test but negative at the preceding donation. Thus, the infection with HEV must have occurred and cleared within the two-month period between the preceding and the index blood donation.

Collectively, we now report a possible longer-term persistence of IgM antibodies after HEV exposure, even after viral clearance.

## 4. Discussion

Most cases of HEV genotype 3 infection are asymptomatic, and the majority of people achieve spontaneous clearance of the virus. Indeed, Faber et al. have previously reported a seroprevalence of anti-HEV IgG of 16.8% in the adult German population, and this seroprevalence increased with age [11]. Our results from 1000 blood donors are in line with those findings, with an overall seroprevalence of 16.6%. Of note, over 30% of people over 55 had HEV antibodies, suggesting a high lifetime infection risk. Notably, Hepatitis E has been reported to be transmitted by blood transfusion [3,4]. For this reason, all blood donors in Germany are tested for HEV by PCR. All individuals in the present cohort were negative for HEV-RNA by PCR, as tested in pools of 24 donor samples (LOD of 240 IU/mL). Unpooled testing of individual blood donations by PCR would increase the sensitivity of the PCR test and thus make it possible to identify very low-viremic individuals. In our study, low-level viremia in preceding or index donations may have been missed due to the insufficient sensitivity of testing in pools of 24 donor samples. Therefore, we recommend performing single PCR testing in doubtful cases in similar future studies. At present, it remains unclear whether this occurrence would have an impact on our particular study setting, and further studies are needed to evaluate this.

Surprisingly, we found 13 people with positive anti-HEV IgM, indicating a recent HEV infection, despite PCR negativity, suggesting rapid clearance of viremia, followed by rapid development of anti-HEV IgM in these asymptomatic blood donors. By analyzing the preceding and follow-up samples of these individuals, we were able to report persistence of IgM antibodies over a period of at least 4–7 months. In three of these donors (donors 3, 5, and 6), we were able to demonstrate fluctuating anti-HEV IgM OD values (Figure 2), but at almost the same level, indicating the persistence of anti-HEV IgM and not re-exposure. It should be noted that this anti-HEV IgM assay is not standardized for quantitative statements. However, three individuals are not sufficient to draw valid conclusions regarding this topic; larger studies are needed. It would certainly have been better to analyze the “sample-to-cutoff ratio values” instead of the OD values to determine the amount of anti-HEV IgM semi-quantitatively, but unfortunately this became clear only during the review process for this project, and at that point the ELISA cutoff values for the individual runs were no longer available. We strongly recommend that this be taken into account in future studies.

In a Spanish pilot study of 25 patients with true acute hepatitis E and 50 blood donors with asymptomatic HEV infection, with a median follow-up of 34 months and using two IgM assays, it was shown that anti-HEV IgM was detectable in 80–100% of patients in the second year after infection and in 17–42% in the third year [12]. Among the 50 viremic blood donors, approximately 25% tested positive for anti-HEV IgM. Unfortunately, it was not possible to analyze the long-term course for this subgroup. We were able to obtain that missing information through our study. Thus, our data demonstrate for the first time that anti-HEV IgM positivity in a European HEV genotype 3 endemic area might not indicate a very recent HEV infection but one that could have occurred several months prior to the serological test in healthy, asymptomatic blood donors. This observation in blood donors from Germany is in line with previous observations in Chinese patients with acute hepatitis E [13,14]. European studies have also shown that the duration of persistence of anti-HEV IgM in the post-viremic period after acute HEV infection varies according to the assay used [12,15,16]. Both inter-assay variability and immunological differences between regions with different endemic genotypes, as well as between patients with genuine hepatitis E and asymptomatic blood donors with HEV, require further consideration in future studies.

Furthermore, there was a relevant correlation between IgG and IgM OD values in our study. However, it is too early to draw valid conclusions regarding a temporal relationship between anti-HEV exposure and anti-HEV IgG titers in asymptomatic blood donors, and the qualitative serological assay used was not designed for quantitative conclusions based on OD values. Future studies should longitudinally investigate the avidity and affinity of anti-HEV antibodies. By examining affinity (strength of a single molecular bond) and avidity (sum of the strengths of several individual bonds), it is possible to draw conclusions about the time since infection and, if this is known, about the strength of the persistent humoral HEV-specific immune response.

In two anti-HEV IgM-positive individuals (Individuals 7 and 9, Table 1), anti-HEV IgG seroconversion did not occur during follow-up. It remains unclear whether a false-positive anti-HEV IgM test or a lack of IgG production following a short exposure of the immune system to HEV explains this finding. The other individuals showed expected anti-HEV IgG seroconversion, and, in those individuals, we also observed the long-term persistence of IgM antibodies after viral clearance. Future studies should use various anti-HEV IgM assays to rule out false positivity in prospective longitudinal studies.

However, the likelihood of false positivity should not be overestimated. A recent large meta-analysis of 21 studies with more than 8000 blood samples found that anti-HEV IgM assays have a specificity of 98% and a sensitivity of 83%, and that the Wantai IgM test we used was the most accurate [17]. One should bear in mind that such meta-analyses and studies evaluating the value of anti-HEV IgM assays are concerned with investigating real hepatitis E and not asymptomatic HEV infection, as we do.

In patient groups, especially immunosuppressed patients, non-specific cross-reactivity or mimicry phenomena can certainly occur more frequently than in blood donors without a relevant pre-existing disease. However, since the rate of false-positive results in asymptomatic blood donors with HEV is unknown, it is not possible to estimate the extent to which false-positive anti-HEV IgM results occur and influence this study. However, in a cohort of 153 patients with acute hepatitis of unknown origin, it has already been shown that false-positive anti-HEV IgM results can occur in cases of acute hepatitis caused by CMV or EBV [18]. Another study of a retrospective cohort of 1423 patients from London who had been tested for anti-HEV IgM showed that 2% tested positive for anti-HEV IgM. Even in those subjects in this study who were HEV PCR-negative, EBV and CMV infections could be identified as the most likely cause of a false-positive anti-HEV IgM test [19]. Since cross-reactivity means that approximately one third of patients who test positive for anti-HEV IgM also test positive for anti-CMV or anti-EBV IgM, it is important to be aware that false-positive anti-HEV IgM results do play a significant role in patients with hepatitis [20]. The extent to which this affects asymptomatic blood donors, as in our study, remains unclear for the time being. One important limitation of our study is that we did not test the anti-HEV IgM-positive results for anti-EBV IgM and anti-CMV IgM.

However, his study demonstrates that anti-HEV IgM positivity can persist for more than six months in blood donors who had neither clinically overt hepatitis E nor a viremia duration that would allow PCR positivity to be detected in consecutive blood donations.

## Figures and Tables

**Table 1:** Serology results of the index, preceding and follow-up blood donation of 13 individuals with positive anti-HEV IgM serology at the index blood donation.

	Preceding Blood Donation			Index Blood Donation			Follow-Up Blood Donation		
Individual #	Date	Anti-HEV		Date	Anti-HEV		Date	Anti-HEV	
		IgG	IgM		IgG	IgM		IgG	IgM
1	n.a.	n.a.	n.a.	20 March 2020	+	+	12 June 2020	+	+
2	n.a.	n.a.	n.a.	19 March 2020	+	+	15 May 2020	+	Borderline
3	13 November 2019	+	+	17 March 2020	+	+	15 June 2020	+	+
4	10 January 2020	-	-	16 March 2020	+	+	25 May 2020	+	+
5	13 January 2020	+	+	16 March 2020	+	+	11 May 2020	+	+
6	19 December 2019	+	+	17 March 2020	+	+	2 June 2020	+	+
**7**	**17 December 2019**	**-**	**-**	**17 March 2020**	**-**	**+**	**14 July 2020**	**-**	**Borderline**
8	21 January 2020	+	+	17 March 2020	+	+	12 May 2020	+	Borderline
**9**	**27 December 2019**	**-**	**-**	**20 March 2020**	**-**	**+**	**12 June 2020**	**-**	**-**
10	n.a.	n.a.	n.a.	19 March 2020	+	+	n.a.	n.a.	n.a.
11	n.a.	n.a.	n.a.	23 March 2020	+	+	n.a.	n.a.	n.a.
12	n.a.	n.a.	n.a.	25 March 2020	+	+	n.a.	n.a.	n.a.
13	n.a.	n.a.	n.a.	26 March 2020	-	+	n.a.	n.a.	n.a.

Patients 7 and 9 with unusual isolated positive IgM findings are printed in bold.

## Data Availability

Access to all original data can be granted on request to the corresponding author.
